# Chiari 1 Malformation, Factors That May Influence Decision Making, and Introducing the Chiari 1 Malformation Severity Classification System

**DOI:** 10.3390/jcm14176113

**Published:** 2025-08-29

**Authors:** Stuart Holder, Muath Abdelkarim Abbakr, Amelia Haynes, Taofiq Desmond Sanusi

**Affiliations:** 1 Department of Neurosurgery, Essex Neurosciences Centre, Queens Hospital, Romford RM7 0AG, UKdesmond.sanusi@nhs.net (T.D.S.); 2 Department of Neurosurgery, University Hospital Southampton, Southampton SO16 6YD, UK

**Keywords:** Chiari 1 malformation, foramen magnum decompression, cerebellar tonsillar descent, syrinx, surgical indications, neurosurgery

## Abstract

**Background/Objectives**: Chiari 1 malformation (CM-1) is a structural abnormality characterised by cerebellar tonsillar descent of 5 mm or more through the foramen magnum. Despite improved imaging, surgical criteria remain inconsistent. This study evaluates the correlation between classical symptoms, imaging findings, and need for surgical intervention, as well as introducing a novel Chiari 1 scoring system aimed at refining the criteria for management options. **Methods**: A retrospective study was conducted on adult patients who were evaluated for CM-1 at a tertiary neurosurgical department with a specialised Chiari and Syringomyelia service over 12 months. Data included demographics, symptoms, imaging characteristics, and surgical intervention. Statistical analysis was performed using SPSS Version 30. **Results**: Sixty-nine (69) patients met the inclusion criteria (mean age, 36.26 years; male-to-female ratio, 1:3.1). Thirty patients (43.5%) had classic symptoms, of whom 26 had a tonsillar descent of >10 mm. A significant association was noted between surgery and classic symptoms (*p* < 0.01), tonsillar descent of >10 mm (*p* < 0.01), and syrinx presence (*p* = 0.02). Our novel scoring system had an AUC of 0.974 (95% CI 0.94–1.00; *p* < 0.001), with an optimum cut of the value of ≥3 points leading to a sensitivity of 100%, specificity of 89.5%, positive predictive value of 66.7% and negative predictive value of 100%. **Conclusions**: Surgical intervention remains an effective option for symptomatic patients. Our novel scoring system could provide a simplified, practical, and more accurate method for identifying patients who may benefit from surgical intervention.

## 1. Introduction

Chiari 1 malformation (CM-1) is a congenital or acquired hindbrain abnormality characterised by the downward displacement of the cerebellar tonsils by 5 mm or more through the foramen magnum [1,2]. It has a reported prevalence of 3% of the population, with approximately one-third of patients becoming symptomatic [3]. This structural alteration can lead to significant cerebrospinal fluid (CSF) flow obstruction and compression of neural structures, resulting in a wide range of neurological symptoms.

The aetiology of CM-1 remains incompletely understood. Although numerous hypotheses have been proposed, no singular theory can account for all cases or the associated abnormalities [4]. These proposed mechanisms range from developmental malformation during the embryonic period to acquired hydrodynamic or structural factors.

From an embryological perspective, Hans Chiari was one of the first to document an embryological theory suggesting that inadequate bony growth of the occiput could lead to overcrowding of the hindbrain and subsequent elevation in intracranial pressure, resulting in tonsillar herniation [5]. This idea was supported by research almost a century later, when the volume of the posterior fossa was found to be proportionally smaller in patients with CM-1 than in the control groups, with a resultant smaller volume correlating to more significant tonsillar herniation [6]. Furthermore, experimental studies conducted by Marín-Padilla induced mesodermal insufficiency in animal embryos via teratogens such as vitamin A, which disrupted occipital somite development and posterior fossa formation, resulting in hindbrain herniation resembling Chiari malformation [7]. This further supported an embryological origin for CM-1. Although a small posterior fossa is not present in all patients with CM-1, those with a small posterior fossa tend to be symptomatic at an earlier age, present with syringomyelia, and exhibit a better response to suboccipital decompression [8,9,10,11].

**Figure 1:**
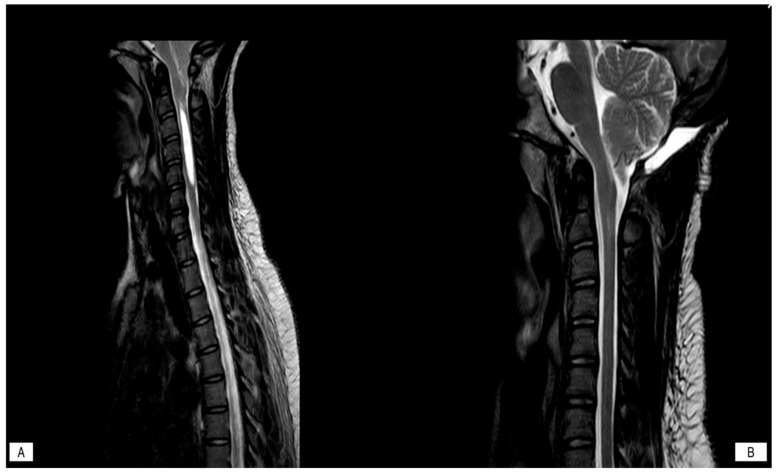
Demonstrating resolution of syrinx and significant CSF flow at the craniocervical junction. (**A**): pre-operative Sagittal T2 weighted imaging; (**B**): Post-operative Sagittal T2 weighted imaging.

While earlier hypotheses focused on embryological or hydrodynamic processes, there has been growing evidence of genetic contributions to Chiari 1 malformations. Familial clustering of Chiari has long been observed, but recent studies have begun to identify specific genetic markers. Rodríguez-Blanque et al. in 2023 conducted a systematic review of Chiari epidemiology and noted an emerging consensus that a genetic basis for the syndrome may exist, at least in some patients [12]. A 2019 study by Musolf et al. linked familial CM-1 with quantitative trait loci on chromosomes 1q43, 1q44 and 12q23, regions that may influence posterior fossa development [13]. Additionally, disorders of connective tissue, such as Ehlers–Danlos and Marfan syndromes, are overrepresented in Chiari cohorts, suggesting that genes affecting collagen, bone, or dural elasticity can predispose to tonsillar herniation [14]. A recent review on the genetics of CM-1 by Yan et al. in 2023 proposed that Chiari 1 malformation cases can be grouped into three broad genetic subclasses: (1) posterior fossa hypoplasia-linked Chiari, probably due to genes that cause underdevelopment of the skull base, (2) macrocephaly-linked Chiari, where an enlarged brain or hydrocephalus due to a genetic syndrome pushes the tonsils down, and (3) connective tissue disorder-linked Chiari, where hypermobile craniovertebral joints or soft dura allow the cerebellum to slip downward [15]. These genetic perspectives were absent from the older literature but have become a focus in recent decades. While the genetics of CM-1 are still being unravelled, the paradigm is shifting from treating Chiari as a singular malformation to a heterogeneous condition with distinct subtypes and aetiologies. Notably, in 2022, Ciaramitaro et al. convened an international consensus and concluded that genetic factors and connective tissue abnormalities contribute to many adult Chiari cases; meanwhile, research continues [16] (Figure 1).

In the evaluation of patients with CM-1, radiological studies are crucial. The primary radiological criterion for diagnosing Chiari 1 is cerebellar tonsillar descent ≥5 mm below the foramen magnum in adults [1,2]. This MRI finding defines the malformation, but it does not dictate management by itself, as evidenced by the fact that as many as 1 in 100 people may have more than 5 mm tonsillar herniation incidentally on MRI. In contrast, only 1 in 1000 patients have symptomatic CM-1 [17]. Thus, the degree of tonsillar descent should be interpreted cautiously and evaluated in conjunction with clinical signs and symptoms.

One important radiologic tool is cine phase-contrast MRI, which visualises CSF flow at the craniocervical junction. Cine MRI was hoped to predict which Chiari patients needed surgery by demonstrating impaired CSF pulsatile flow. Early studies suggested that absent or markedly reduced CSF flow posterior to the tonsils correlated with symptoms and the presence of a syrinx and that normalisation of flow post-operatively correlated with relief. However, a 2021 systematic review in the Congress of Neurological Surgeons (CNS) guidelines found that evidence was insufficient to say cine MRI reliably predicts surgical benefit. They gave only a Grade C recommendation, stating that cine MRI may or may not predict benefit from surgical decompression, reflecting mixed results in the literature [18]. In practice, many adult CM-1 patients do require MRI flow studies. If a complete CSF obstruction is observed, i.e., no flow ventrally or dorsally in systole or diastole, this may tip the scales towards surgical intervention, especially if symptoms align. However, the presence of flow does not exclude symptomatic Chiari. Thus, cine MRI could be a helpful adjunct but not an absolute criterion.

Other radiographic criteria include the presence of a syrinx and measurements of craniovertebral alignment. Syringomyelia on spinal MRI is a pivotal factor. The literature agrees that a syrinx in the context of a CM-1 is an indication for strong consideration of decompression due to the risk of permanent neurological damage [19]. This is described by Massimi et al. in 2019, who found significant improvement both clinically and radiologically in patients who underwent surgical intervention [20].

The criteria for surgical intervention are nuanced and depend on both clinical and radiological findings. Patients who experience the classic constellation of symptoms, occipital headaches exacerbated by Valsalva manoeuvres, neck pain, lower cranial nerve signs, cerebellar ataxia, or myelopathic symptoms from associated syringomyelia, are typically considered for surgery [21]. These symptoms indicate significant neural compression or cerebrospinal fluid (CSF) flow obstruction at the craniocervical junction. The World Federation of Neurosurgical Societies (WFNS) guidelines strongly recommend a symptom-based approach for surgical intervention in patients with CM-1 [21].

Conservative management should be the default approach for asymptomatic patients. Non-operative management is supported by natural history studies, which show that many asymptomatic or mildly symptomatic adults remain stable or even improve spontaneously over time [22,23]. However, some clinical scenarios in asymptomatic or minimally symptomatic patients may warrant surgical consideration, particularly in cases of syringomyelia. Even if headaches or neurological deficits are mild, large and symptomatic syringomyelia could require surgical intervention in the form of foramen magnum decompression. This could prevent clinical deterioration or lead to symptomatic improvement [19]. In addition, the progression of high-risk features, such as increasing syrinx size, new onset hydrocephalus, or worsening craniocervical angulation, on serial radiological imaging can also be an indication for surgery in an asymptomatic adult [21].

**Figure 2:**
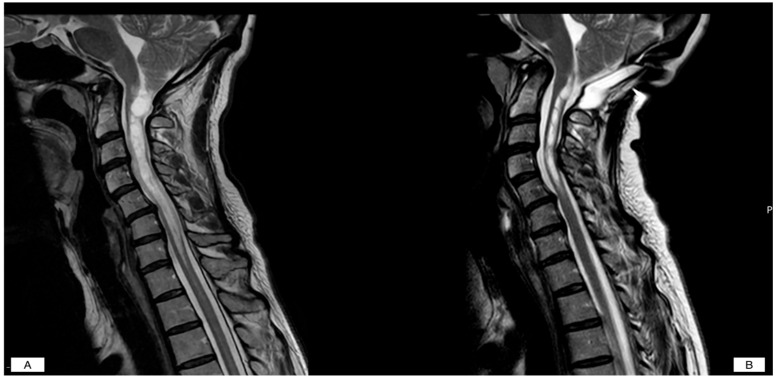
Mild clinical CM-I symptomatology with symptomatic syringomyelia. (**A**): Pre- and post-operative MRI scans demonstrating a significant reduction of the syrinx. (**B**): Post-operative MRI was obtained after 6 months.

Several investigative groups have attempted to introduce objectivity into the diagnosis, decision-making and post-operative evaluation of CM-I. The Chicago Chiari Outcome Scale (CCOS) quantifies four post-operative domains (pain, non-pain symptoms, functionality, complications) on a 16-point scale. It reliably distinguishes between improved and unimproved patients [24]. More recently, the Chiari Severity Index (CSI) incorporated pre-operative clinical and imaging variables to predict the likelihood of long-term quality-of-life gain after surgery; however, this was in the paediatric population [25]. While these tools are valuable in clinical practice and research, they do not provide a concise bedside rule for decision-making (Figure 2).

Our Chiari-I Malformation Severity Classification System, CM-I SCS, was developed to help improve pre-operative decision-making and post-operative outcomes. It assigns easily recalled points to the two domains that consistently influence operative benefit and risk, as follows:

- Clinical symptoms—graded by the impact of headache and neurological findings.
- Radiological factors—degree of tonsillar descent with or without syringomyelia.
- The composite score stratifies patients into management pathways that range from conservative management to surgical recommendation. This could help to standardise practice across providers and institutions.

By providing an integrated clinical–radiologic score that correlates directly with treatment advice, CM-I SCS aims to improve consistency in counselling, facilitate prospective audits of surgical outcomes, and furnish a common language for multicentre trials.

## 2. Materials and Methods

This retrospective cohort study was conducted at a tertiary neurosurgical department at Queen’s Hospital, Barking, Havering, and Redbridge University Hospital, Greater London, with an established Chiari and syringomyelia service. We assessed patients diagnosed with CM-1 who attended our tertiary, super-specialised Chiari and syringomyelia outpatient clinic between March 2024 and May 2025.

The study included patients aged 18 years or older with radiologically confirmed CM-1, defined as cerebellar tonsillar descent of 5 mm or more below the foramen magnum. Exclusion criteria comprised of patients who had previously undergone foramen magnum decompression, those with incomplete clinical records, or those suffering from concurrent neurosurgical conditions that could confound symptomatology.

Demographic data, including age, gender, medical history, clinical presentations detailing the range and severity of symptoms, imaging findings based on MRI characteristics, and details regarding surgical interventions, were meticulously recorded.

Classic symptoms, defined as headaches associated with Valsalva manoeuvres, temperature disturbances, dizziness, and balance issues, were documented. MRI findings were analysed to determine the degree of tonsillar descent as well as the presence and extent of syringomyelia. Surgical intervention was mainly foramen magnum decompression, C1 laminectomy, and duraplasty. Post-surgical follow-up occurred an average of 4 months after the procedure.

The variables included in the CM-I SCS were selected based on their known or observed correlation with the need for surgical intervention in CM-1. We specifically focused on factors that showed a significant association with the decision to recommend surgery, namely the presence of classic Chiari symptoms, tonsillar descent >10 mm, and presence or absence of syringomyelia (Table 1 and Table 2). To evaluate the scoring system’s performance, we generated a receiver operating characteristic (ROC) curve. The ROC analysis assessed how well the composite score discriminated between patients who required decompression and those managed conservatively. We determined the optimal cutoff score by maximising the Youden index. This approach yielded an optimal threshold of ≥3 points for recommending surgery, which provided the best balance of high sensitivity and specificity in our cohort. We also calculated the area under the ROC curve (AUC) as a measure of the score’s overall diagnostic accuracy in predicting surgical need. Further to this, we then assessed the patient’s outcome using the CCOS.

All statistical analyses were carried out using SPSS Version 30. Descriptive statistics were used to summarise patient characteristics and outcomes. Chi-square and Fisher’s exact tests were conducted to evaluate associations between categorical variables. A *p*-value of less than 0.05 was deemed statistically significant.

## 3. Results

### 3.1. Patient Characteristics

A total of sixty-nine patients with Chiari 1 malformation were included in the analysis. The mean age was 36.26 ± 12.2 years (median 35.5, inter-quartile range 26.5–42.5). The majority were female (52 patients, 75.4%), with seventeen males (24.6%). Classic Chiari symptoms were present in thirty patients (43.5%) at the time of presentation.

All patients had at least 5 mm of tonsillar descent; 26 patients (37.7%) had tonsillar descent of >10 mm. An associated syrinx was noted in eleven patients (15.9%). Twelve patients (17.4%) were recommended for surgical intervention, while 55 (82.6%) were recommended for non-surgical management. Three patients who were recommended for surgery preferred not to undergo an operation despite progressive symptomatology.

### 3.2. Bivariate Associations

On univariate analysis, age and sex were not found to be significantly associated with whether patients underwent surgical decompression. The median age was similar in both the surgical and non-surgical groups. The surgical recommendation rate was 11.7% in males (2 of 17) versus 23.0% in females (12 of 52). However, this difference was not statistically significant (*p* = 0.481), indicating that patient sex did not affect the treatment decision; the sample size may also have been a contributing factor.

Clinical presentation and radiographic features showed significant associations with surgical intervention. Among the thirty patients with classic Chiari symptoms, 11 (36.6%) were recommended for surgical decompression, compared to only 1 of 39 patients (2.85%) without classic symptoms. This difference was highly significant (*p* < 0.001), indicating that patients presenting with typical Chiari 1 symptoms were significantly more likely to undergo surgical treatment.

A syrinx on imaging was also associated with a higher likelihood of surgery. 5 of 11 patients (45.5%) with any syringomyelia underwent surgery, versus 7 of 58 (12.0%) without a syrinx.

The degree of tonsillar descent was another important factor. Patients with tonsillar descent of >10 mm had a significantly higher recommendation rate for surgery than those with tonsillar descent of ≤10 mm. Specifically, 11 of 15 patients (73.3%) with a descent of >10 mm were recommended for foramen magnum decompression, compared to 1 of 42 (2.3%) with a descent of ≤10 mm (*p* < 0.001).

In summary, the univariate analyses revealed that patients were more likely to be recommended for surgery if they presented with classic symptoms, had tonsillar descent of more than 10 mm, or had a syrinx present. Age and gender did not influence surgical recommendations.

### 3.3. Diagnostic Performance of the Scoring System

This novel Chiari 1 Malformation Severity Classification System, incorporating stratified clinical and radiological factors, showed a strong relationship with treatment outcomes. The total Chiari 1 score in our cohort ranged from 0 to 4 points. Patients who underwent surgery had markedly higher composite scores than those managed conservatively. The score was strongly associated with management, as indicated by chi-square analysis (*p* < 0.001), confirming that higher scores were observed in the surgical group.

Using the CM-I SCS to predict the need for decompression, we identified an optimal cutoff value of 3 points or greater for a positive test. This threshold was chosen as it provided the best balance of sensitivity and specificity, maximising the Youden index. At a score of 3 or higher, the sensitivity for correctly identifying patients who would benefit from surgery was 100%, and the specificity for correctly excluding those who did not require surgery was 89.5%. The positive predictive value (PPV) of a score of 3 or higher was 66.7%, indicating that two-thirds of patients meeting this threshold were actual surgical cases. The negative predictive value (NPV) was 100%, indicating that patients with scores below three were correctly managed without surgery almost 90% of the time. Notably, a higher cutoff of 4 points achieved 100% specificity, but at the cost of significantly lower sensitivity, 50%, missing most surgical cases. In contrast, a lower cutoff (≥2) improved sensitivity to 100% but reduced specificity to 66.7%, resulting in a higher number of false positives. Thus, a threshold of 3 points was deemed optimal for the diagnostic performance of this scoring system.

### 3.4. ROC Curve Analysis

**Figure 3:**
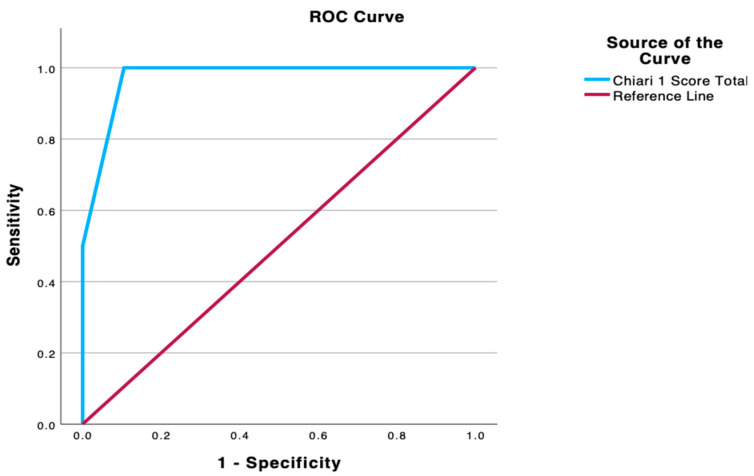
Receiver operating characteristic.

ROC curve analysis further demonstrated the discriminative ability of the CM-I SCS. The ROC curve lay well above the diagonal no-discrimination line, reflecting good test accuracy. The AUC was 0.974 (95% CI, 0.94–1.00; *p* < 0.001), indicating a strong overall ability of the score to distinguish between patients who required surgery and those managed non-surgically. The ROC analysis confirmed a total score of 3 as the optimal cutoff for predicting the need for surgical decompression. This cutoff yielded the highest combination of sensitivity and specificity, as noted above, and thus maximised the correct classification of patients. These ROC findings indicate that this novel scoring system has good predictive value, and a score of 3 points or higher can be justified as the decision threshold for recommending surgical intervention in patients with CM-1 (Figure 3).

## 4. Discussion

The new integrated Chiari 1 Malformation Severity Classification System demonstrated strong performance in distinguishing between patients who underwent surgical management and those who received conservative management in our cohort. In our cohort of sixty-nine adults with CM-1 (mean age, 36.26 years; 75.4% female), univariate analysis revealed that factors such as classic Chiari symptoms, tonsillar descent exceeding 10 mm, and syringomyelia were all predictors of surgical intervention. 36.6% of patients with Valsalva-associated headache or other classical symptoms underwent surgery, compared to only 2.85% of those without (*p* < 0.001). Additionally, those with tonsillar descent greater than 10 mm were more likely to undergo surgery than those with a descent of ≤10 mm (73.3% vs. 2.3%, *p* < 0.001).

We chose to distinguish between the extent of the syringomyelia in our scoring scheme, even though in this study any syrinx, regardless of length, was managed similarly. However, we maintained this 1A/1B distinction because it may prove valuable in future research evaluating the difference in clinical outcomes or recovery patterns depending on the extent of the syrinx, similar to the paediatric population [26].

Using ROC analysis, a score threshold of ≥3 optimally balanced sensitivity (100%) and specificity (89.5%) for predicting the decision to decompress. The area under the ROC curve was 0.974 (95% CI, 0.94–1.00; *p* < 0.001), indicating good overall discrimination of the scoring system. In our cohort, a cutoff of ≥3 yielded a high negative predictive value (100%), meaning that patients with scores below this threshold were correctly managed non-operatively nine out of ten times. These results suggest that the score has meaningful predictive power. A very high cutoff (score = 4) achieved perfect specificity but poor sensitivity (50%), while a low cutoff (≥2) yielded high sensitivity (100%) but poor specificity (66.7%). This suggests that the three thresholds offer the optimal trade-off for clinical decision-making.

The proposed score must be considered in the context of CM-1 grading and outcome tools. The Chicago Chiari Outcome Scale is a well-known 16-point outcome instrument that grades post-operative status in four domains [27]. In its development, patients with CCOS 13–16 were judged as improved, whereas those with 4–8 were noted to be worse. External validation confirmed the CCOS’s utility for standardising outcome assessment, with excellent discrimination, AUC ≈ 0.95, for identifying improved patients [24]. However, the CCOS is not a pre-operative stratification tool and requires detailed post-operative follow-up, limiting its use in surgical decision-making.

The Chiari Severity Index (CSI) is the closest analogue to our grading system. Greenberg et al. developed the CSI as a pre-operative index of patient improvement in the paediatric population, integrating headache/myelopathy symptoms and the presence of a large syrinx to predict post-operative quality-of-life benefits [25]. The CSI stratified patients into three grades, with improvement rates ranging from 83% (grade 1) to 45% (grade 3) (*p* = 0.002), and its overall discrimination was moderate. In practice, the CSI assists with counselling but categorises risk broadly rather than providing a binary recommendation. However, our score uses clinical indicators and simple radiologic cutoffs to yield a straightforward numeric result. Our data yielded an AUC of 0.974 for predicting surgeon recommendation, which compares favourably to the CSI’s reported improvement in prediction. It is also worth noting that, in post-surgical follow-up, eight of the nine patients had a CCOS score of 16, while one had a CCOS score of 12.

Other stratification schemes have emphasised craniovertebral junction (CVJ) morphology. For example, He et al. proposed a CVJ compression severity index that categorises patients with CM-1 by ventral, dorsal, or combined brainstem compression [28]. They defined six grades, with higher grades correlating with more severe craniocervical deformity and worse outcomes [28]. The craniovertebral junction compression severity index highlights the role of ventral compression, but it is complex to calculate and does not incorporate symptom burden.

Similarly, CVJ alignment measures have been used to influence decision making. The clivo-axial angle (CXA) and the posterior basion to C2 (pB-C2) distance quantify skull-base flexion and odontoid projection, respectively. The CNS 2021 guidelines note that measuring CXA, pB-C2, or C1–C2 alignment may help to predict future craniocervical instability and the need for stabilisation. However, this is a weak Grade C recommendation, as the evidence is limited, but it highlights a change observed over the past decade [18].

In contrast, Goel et al. [29] hypothesised that subtle atlantoaxial instability (AAI) at C1–C2 underlies many adult CM-1 cases by allowing cranial settling and driving tonsillar herniation. In Goel’s series, C1–C2 fusion, without suboccipital decompression, resulted in clinical improvement and partially reversed the tonsillar descent; Salunke et al. described similar cases [29,30]. However, most adult CM-1 patients lack radiographic evidence of AAI, and current consensus is that atlantoaxial fusion should be reserved for those with overt CVJ instability coexisting with tonsillar descent [21,31].

In comparison, the new score combines clinical and radiological dimensions into a single index. It also offers the simplicity of a total score while addressing multiple relevant disease aspects, providing more holistic stratification than symptom-only or imaging-only schemes.

There are several advantages to the new scoring system. It enables the integration of clinical and radiological data, considering both symptom severity and imaging findings. This comprehensive approach mirrors the recognised factors that drive management decisions, whereas other systems often emphasise only one domain. The new scoring system is simple to use and is objective. It employs clear binary criteria and a small point scale, yielding a result that is easy to calculate and interpret. Unlike subjective judgment, this score yields explicit guidance. The cutoffs map directly to management while demonstrating good discrimination. Furthermore, by formalising the decision process, the score may reduce variability in counselling and create a common language linking symptoms, imaging, and recommendations. It should be noted as well that since CM-1 is a heterogeneous entity, all patients may not fit precisely into the classification system. An example of such is the subset of patients who may not have classical Chiari symptoms but have severe syringomyelia with crowding of the foramen magnum. These patients may still require surgical intervention once no other cause of their syringomyelia has been identified.

In our series, the alignment between the CM-I SCS recommendations and patient outcomes was encouraging. Eight of nine decompressed patients achieved the maximum CCOS score of 16 at follow-up, indicating excellent post-operative improvement, and the remaining surgical patient had a CCOS of 12, indicating a meaningful improvement. This suggests that patients selected for surgery using our score generally experienced the anticipated benefit, further supporting the score’s validity as a decision tool. In addition, the high predictive accuracy of our scoring system is comparable to the CCOS’s ability to distinguish improved vs. non-improved patients, thus showing that our pre-operative score can achieve discrimination on par with detailed post-operative outcome measures. This reproducibility and congruence with known outcome metrics show that the CM-I SCS is both a valid and a practical tool for CM-1 management.

It should also be recognised that certain anatomical factors outside our scoring parameters can influence management decisions. For instance, craniosynostosis, which is commonly diagnosed in the paediatric population, can reduce posterior fossa volume and precipitate hindbrain crowding. However, we believe that our scoring system would be adaptable to the paediatric population with the modification of the clinical presentations, the other parameters of the scoring system would be similar to adults. In addition, CM-1 patients with concurrent raised intracranial pressure or hydrocephalus should be evaluated and will require intervention prior to consideration for posterior fossa decompression if indicated [32]. Thus, identifying these conditions preoperatively is crucial. In the presence of craniofacial abnormalities, CT scans with 3D reconstruction should be obtained to further evaluate this subset of patients and referral made to a craniofacial specialist for evaluation [33].

Overall, our new scoring system parallels the intent of other standardised tools. Once validated, such a score can support prospective audits and multicentre studies by ensuring that clinical severity is categorised uniformly.

## 5. Limitations

Our study’s limitations should be acknowledged. First, this is a retrospective, single-centre study with a modest sample size. However, this was conducted over a period of one year. A larger sample size would be needed to confirm reproducibility. In addition, our study did not include the paediatric population. Finally, no scoring system can capture all aspects of Chiari 1 pathology. Our score does not incorporate dynamic CSF flow studies or other subtleties, so that some nuanced cases may fall outside the criteria. However, we believe these investigations would add credence to the scoring system.

## 6. Conclusions

The Chiari 1 Malformation Severity Classification System has proven to be a simple and objective tool for determining which patients may benefit from surgical intervention. With a cutoff ≥ 3, it achieved 100% sensitivity, 89.5% specificity, and an AUC of 0.974. The ease of use and clarity may enhance consistency in patient counselling and facilitate a multicentre collaboration through standardised severity grading. Although these results are derived from a single-centre, retrospective adult cohort, they highlight the score’s utility in surgical decision-making. We are in the preliminary stage in organising a multicentre prospective study. This will aid in confirming the score’s generalizability, interobserver consistency, and determine if any adjustments are required for different populations.

## Figures and Tables

**Table 1:** The Chiari 1 Malformation Severity Classification System, CM-I SCS.

Component	Criteria	Score
Clinical Symptoms	Non-classical Chiari headache or non-specific headache	0
	Mild (Intermittent, not affecting functionality) Classical tussive headache Occipital headache with generalisation Neck pain	1
	Moderate (Functional limitation) Classical tussive headache Occipital headache with generalisation Neck pain Nausea and vomiting Signs of cord compression ○ Balance issues ○ Dizziness	2
	Severe (Functional limitation) Classical tussive headache Occipital headache with generalisation Neck pain Signs of cord compression ○ Balance issues ○ Dizziness Drop attacks Autonomic dysfunction Lower cranial nerve dysfunction	3
Tonsillar Descent on MRI (mm)	5–10 mm below the foramen magnum	0
	>10 mm below the foramen magnum	1
Extent of Syrinx on MRI	Absent	0
	Cervical only	1A
	Cervical and/or thoracic	1B

Tonsillar ectopia that has been described in cases such as Chiari 0 or Chiari 0.5 is outside the scope of this scoring system. However, this scoring system can still be applicable in this cohort of patients, depending on the clinical presentation and the presence of a syrinx.

**Table 2:** The CM-I SCS grades and recommendations.

Grade	Recommendation
0	Reassurance and no follow-up needed
1	Conservative management and consider no follow-up
2	Conservative management and consider interval follow-up (if indicated)
3	Equipoise: Option for surgery or conservative management
4	Recommend surgical intervention
5	Recommend surgical intervention

Emphasis could be given to the presence of syringomyelia in the absence of classical headache if there is tonsillar herniation with crowding at the foramen magnum.

## Data Availability

The data presented in this study are available on request from the corresponding author.
